# Green Pre-Treatment Strategy Using Ionic Liquid-Based Aqueous Two-Phase Systems for Pesticide Determination in Strawberry Samples

**DOI:** 10.3390/foods13244106

**Published:** 2024-12-18

**Authors:** Ana Jocić, Slađana Marić, Danijela Tekić, Jasmina Mušović, Jelena Milićević, Sanja Živković, Aleksandra Dimitrijević

**Affiliations:** 1 Department of Physical Chemistry, VINČA Institute of Nuclear Sciences—National Institute of the Republic of Serbia, University of Belgrade, Mike Petrovića Alasa 12-14, 11351 Belgrade, Serbia; ana.jocic@vin.bg.ac.rs (A.J.); danijela.tekic@vin.bg.ac.rs (D.T.); jasmina.musovic@vin.bg.ac.rs (J.M.); sanjaz@vin.bg.ac.rs (S.Ž.); daleksandra@vin.bg.ac.rs (A.D.); 2 Department for Bioinformatics and Computational Chemistry, VINČA Institute of Nuclear Sciences—National Institute of the Republic of Serbia, University of Belgrade, Mike Petrovića Alasa 12-14, 11351 Belgrade, Serbia; jdjordjevic@vin.bg.ac.rs

**Keywords:** aqueous two-phase system, ionic liquid, pesticide in food, preconcentration, strawberry, clomazone, pyraclostrobin, deltamethrin

## Abstract

Pesticides used in agriculture can contaminate foods like fruits and vegetables, posing health risks to consumers and highlighting the need for effective residue monitoring. This study explores aqueous two-phase systems (ATPSs) comprising phosphonium or ammonium ionic liquids (ILs) combined with ammonium sulfate as an alternative pre-treatment method for extracting and concentrating the pesticides clomazone, pyraclostrobin, and deltamethrin from strawberry samples. Liquid–liquid equilibrium measurements for each ATPS were conducted, followed by extraction experiments to determine the most efficient systems for pesticide extraction. Results showed that all three pesticides migrated effectively to the IL-rich phase across the tested ATPSs. For the most promising system, tetrabutylphosphonium salicylate ([TBP][Sal]) with ammonium sulfate, extraction efficiencies for each pesticide exceeded 98% under optimized conditions for parameters such as pH, temperature, and ATPS composition. Application of this ATPS to strawberries resulted in significant pesticide preconcentration, reaching mg/L levels suitable for detection by liquid chromatography. The method’s sustainability was supported by green chemistry metrics, with AGREEprep and AGREE scores of 0.68 and 0.55, respectively, underscoring its alignment with eco-friendly practices.

## 1. Introduction

As the global population continues to rise, the demand for increased food production has grown substantially [1]. To meet these demands and protect agricultural products, the widespread use of pesticides has become essential. Without such protective measures, it is estimated that crop yields would only achieve about 30% of their full potential [2]. Despite their essential role, the prolonged use of pesticides has led to environmental pollution and raised significant food safety concerns due to the accumulation of pesticide residues. Even pesticides considered low in toxicity can persist in the environment, leading to pollution of soil and water and threatening non-target organisms and ecosystems. Of particular concern are pesticides, which are suspected of acting as endocrine disruptors, potentially leading to long-term health risks such as hormonal imbalances and reproductive disorders in humans [3,4,5]. These risks underscore the importance of routinely monitoring pesticide residues in food products, especially in fruits and vegetables, which are common sources of exposure. Regulatory bodies, such as the European Commission, have established maximum residue limits (MRLs) for numerous pesticides to ensure safe consumption levels [6]. These regulations help ensure that pesticide levels remain within safe limits, safeguarding public health. However, studies consistently detect residues from pesticides in fruits like strawberries, apples, cherries, and grapes. Strawberries consistently top the ‘dirty dozen’ list, which identifies the 12 fruits and vegetables with the highest pesticide residue levels, alongside apples and grapes [7,8,9]. For instance, a study from Egyptian markets found pesticide residues in all tested fresh strawberries and strawberry-based products, including yogurt, juice, jam, and dried strawberries, with average concentrations ranging from 0.006 to 0.568 mg/kg, depending on the product [10]. Similarly, research from Shanghai on strawberry samples from the 2018 and 2019 harvest seasons revealed 51 pesticide residues, with 97.91% of samples containing at least one pesticide and 2.39% exceeding the maximum residue limits set by Chinese regulations [11]. This underscores the urgent need for reliable, sensitive, and environmentally friendly detection methods for food samples. Detecting pesticide residues accurately is not only essential for adhering to regulatory standards like MRLs but also for minimizing long-term exposure to potentially harmful chemicals. The development of new pesticides and the growing number of pesticide formulations further increase the necessity for ongoing advancements in detection techniques.

Pesticide detection in complex matrices often requires advanced analytical techniques like high-performance liquid chromatography (HPLC) [9,12,13] and gas chromatography (GC), known for their high sensitivity and precision in quantifying trace pesticide levels [4,14,15]. Due to the complexity of matrices like fruits, pre-treatment steps are typically essential to extract analytes effectively and achieve low detection limits. Conventional methods for pesticide preconcentration and extraction, such as solid-phase extraction (SPE) and liquid–liquid extraction (LLE), are effective but come with certain limitations: LLE requires large amounts of organic solvents, while SPE can be labor-intensive. In addition to these techniques, the widely used QuEChERS (Quick, Easy, Cheap, Rugged, Effective, and Safe) method offers a simpler and more efficient approach for pesticide preconcentration, although it predominantly relies on acetonitrile (ACN) as the organic solvent. To mitigate these drawbacks, microextraction techniques like solid-phase microextraction (SPME), liquid-phase microextraction (LPME), and dispersive liquid–liquid microextraction (DLLME) have been developed. These methods are more efficient in minimizing solvent use while still offering effective preconcentration [16,17]. In recent years, ionic liquids (ILs) have emerged as a promising alternative to traditional organic solvents in these extraction processes. Ionic liquids are salts with depressed melting points and possess tunable properties such as low volatility and reduced environmental impact compared to organic solvents. Their tunable nature makes them ideal for optimizing extraction conditions [18,19]. Extensive research has been conducted on the use of ILs in DLLME for pesticide preconcentration, usually from water samples, with acetonitrile as the most common organic solvent. Typically, HPLC is favored over GC in systems involving ionic liquids due to the low volatility of ILs, which complicates GC analysis [20]. As part of the shift toward sustainable solvents, researchers have proposed ionic liquid-based aqueous two-phase systems (IL-ATPSs). These IL-ATPSs, formed by mixing an IL aqueous solution with a salt or polymer solution under controlled conditions (pH, temperature), offer unique advantages, including operational simplicity, low cost, and high biocompatibility. Unlike other methods, which often rely on hazardous organic solvents, ATPSs provide a gentler, predominantly water-based extraction strategy, further enhancing its appeal for environmentally friendly extraction processes. Compared to QuEChERS and DLLME, IL-ATPSs offer a more sustainable and efficient approach to preconcentration. QuEChERS is faster and simpler but has limited preconcentration capacity, while DLLME achieves high enrichment with minimal solvent but suffers from scalability challenges. IL-ATPSs combine the advantages of both, providing high preconcentration efficiency with a greener solvent profile [16,18,21]. Despite the promising benefits of IL-ATPSs, surprisingly few studies focus on their application in the preconcentration of pesticides from food matrices prior to analytical methods. A study by Tian et al., demonstrated the effectiveness of a cholinium acetate ([Ch][Ac]) and EOPO-2500 copolymer IL-ATPS for herbicide extraction from honey, significantly improving sample preparation and preconcentration for accurate HPLC–MS/MS quantification [22]. A similar system, employing EOPO-2500 and cholinium L-lysinate ([Ch][L-Lys]), has been used to extract target fungicides from commercial fruit juice and homemade apple juice. Although this setup showed a modest preconcentration factor, it effectively isolated analytes into the EOPO phase. After phase separation, the enriched phase was analyzed by HPLC-MS/MS, demonstrating reliable quantification of fungicides in complex food matrices [23]. Conversely, ionic liquid and salt-based systems have demonstrated high efficiency and high preconcentration factors in IL-ATPS applications, especially those utilizing ammonium, phosphonium, imidazolium, and cholinium ILs, for the extraction and preconcentration of various contaminants, including pharmaceuticals, bisphenols, and parabens, typically sourced from wastewater samples [24,25,26]. Furthermore, a substantial number of studies have demonstrated the high, often near-complete extraction efficiency of IL-ATPSs for pesticide removal from wastewater. These systems have proven particularly effective as an initial step in sample preparation, concentrating pesticides for subsequent analytical detection. For example, a study using an ATPS composed of choline chloride ([Ch]Cl) and polypropylene glycol 400 (PPG400) demonstrated high extraction efficiencies for pesticides like dicamba, clomazone, pyraclostrobin, and deltamethrin. Nearly complete extraction of these pesticides from agricultural wastewater in one step into the polymer-rich phase highlighted the potential of IL-ATPSs for high-efficiency extraction, with significant preconcentration factors achieved by optimizing the system composition [27]. Additionally, research by Jocic et al. explored the use of tetrabutylphosphonium salicylate ([TBP][Sal]) in combination with a citrate buffer, highlighting its efficacy in the extraction of organophosphate pesticides from wastewater. The study also found that the trace amounts of ionic liquid remaining in the aqueous phase after extraction posed no cytotoxic effects, underscoring the potential of IL-based ATPS as a greener alternative to traditional extraction methods [28]. Given the promising results, this study aimed to evaluate and compare the effectiveness of phosphonium and ammonium ILs in developing an aqueous two-phase system specifically for the preconcentration of pesticides, enabling their detection from complex fruit matrices, such as strawberries. Notably, this research addresses a gap in the literature, as no previous studies have used ionic liquid and salt-based ATPS for this purpose.

In this study, both phosphonium and ammonium ionic liquids with anions derived from natural acids (salicylate, acetate, and lactate) were tested as an alternative pre-treatment strategy for pesticide determination in food samples. Three pesticides from distinct agrochemical groups were selected: clomazone (CLO), a herbicide used for weed control in vegetable crops; pyraclostrobin (PYR), a fungicide targeting fungal infections in plants; and deltamethrin (DLM), a pyrethroid insecticide for pest management [29,30,31]. Initially, phase diagrams for ammonium ionic liquids (TBA-ILs) with ammonium sulfate (as the salting-out agent) were established and compared to those of phosphonium ILs (TBP-ILs). Extraction experiments in the two-phase medium were conducted to identify the most favorable system for the extraction of pesticides. The effects of temperature, pH, and ATPS compositions on the extraction performance of a [TBP][Sal]-based ATPS were systematically investigated. After identifying optimal conditions, the [TBP][Sal]-based ATPS was applied to the preconcentration of pesticides from the strawberry sample, with quantification carried out using the ultra-performance liquid chromatography (UPLC) method. To assess the environmental impact of the proposed method, green analytical metrics AGREEprep and AGREE were utilized. These metrics enabled a comprehensive evaluation of the method’s environmental footprint, from sample preparation through to final analysis, ensuring alignment with the principles of Green Analytical Chemistry [32,33].

## 2. Materials and Methods

### 2.1. Materials

**Figure 1:**
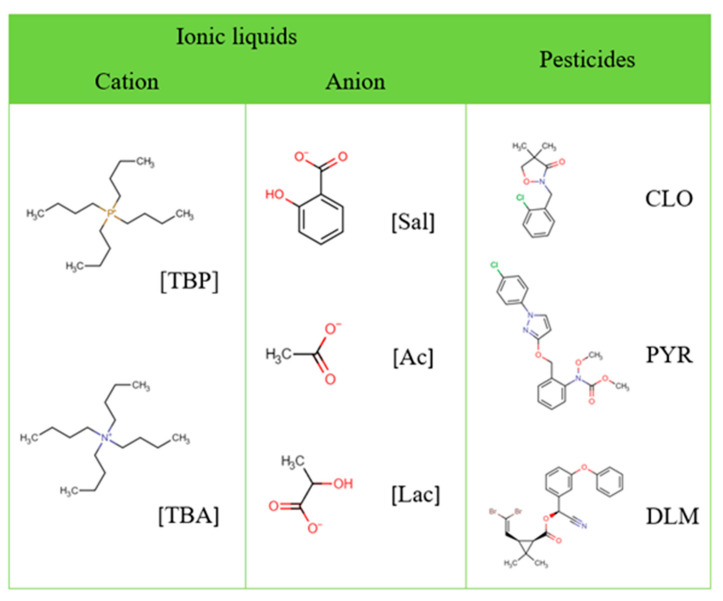
Chemical structures and abbreviations of studied ILs and pesticides.

Three pesticides, namely clomazone, pyraclostrobin, and deltamethrin, were obtained from Sigma-Aldrich (USA), and their chemical structures are shown in Figure 1. HPLC-grade acetonitrile (C_2_H_3_N), ethanol (CH_3_CH_2_OH, ≥99.8 wt%), and acetone (≥99.5 wt%) were procured from Honeywell (Germany). Ammonium sulfate ((NH_4_)_2_SO_4_, ≥99 wt%), tetrabutylammonium hydroxide (C_16_H_37_NO, 40 wt% in H_2_O), and salicylic acid (C_7_H_6_O_3_, ≥99 wt%) were supplied from Sigma-Aldrich (USA), while lactic acid (C_3_H_6_O_3_, ≥90 wt%) was acquired from Fluka Chemie (Switzerland) and acetic acid (CH_3_COOH, ≥99 wt%) was provided by MSK Kikinda (Serbia). Phosphonium ILs, including tetrabutylphosphonium acetate ([TBP][Ac]), tetrabutylphosphonium lactate ([TBP][Lac]), and tetrabutylphosphonium salicylate ([TBP][Sal]) were synthesized in previous study [34]. Ammonium ILs—tetrabutylammonium acetate ([TBA][Ac]), tetrabutylammonium lactate ([TBA][Lac]), and tetrabutylammonium salicylate ([TBA][Sal])—were synthesized through the neutralization of tetrabutylammonium hydroxide with salicylic, acetic, or lactic acid, respectively [35]. The chemical structures of ILs used in this study are provided in Figure 1. The ILs were prepared by mixing equimolar amounts of hydroxide and the corresponding acid, with the mixture being stirred for 24 h at room temperature. Subsequently, water was removed from the obtained ILs under vacuum using a R-210 Rotavapor System BÜCHI Labortechnik AG (Switzerland). Additionally, all ILs underwent a drying process, where they were heated at 50 °C under vacuum for approximately 48 h. Water content was determined via Karl Fischer titration, using an 831 Karl Fischer coulometer from Metrohm, and found to be less than 2 wt% in all ILs. The chemical structures of the ILs were confirmed using a Thermo Fisher Scientific Nicolet iS5 spectrometer (USA), and the corresponding spectra, along with peak assignments, are provided in the Supplementary Materials (Figures S1–S3).

### 2.2. Determination of the Phase Diagrams and Tie-Lines

The liquid–liquid ternary phase diagrams of phosphonium ILs, ammonium sulfate, and water were obtained in previous work using the cloud point titration method at (25 ± 1) °C and atmospheric pressure (0.10 ± 0.01 MPa) [34]. In this study, phase diagrams of ammonium ILs with ammonium sulfate and water were determined using the same method. Briefly, this method involves the dropwise addition of a salt solution (~40 wt%) to a known mass of an ammonium IL aqueous solution (~70 wt%) until the mixture becomes cloudy, followed by the addition of water until the solution becomes clear. After each addition of salt or water, the solution was shaken using a vortex agitator at 2500 rpm (Reax Top, Heidolph), and masses were recorded using an analytical balance (KERN ADJ 210-4) with an uncertainty of 10^−4^ g. This procedure was repeated to obtain enough experimental data points to construct the binodal curve. The experimental obtained binodal curves were fitted by the Merchuk equation [36]:

(1) $$Y=A\;\exp\left[\left({\mathrm{CX}}^{0.5}\right)-\left({\mathrm{BX}}^{3}\right)\right]$$

where Y and X are, respectively, the IL and salt weight percentages; A, B, and C are regression parameters.

Except for the [TBP][Sal]-based ATPS, each tie-line (TL) and tie-line length (TLL) were determined using the gravimetric method described by Merchuk et al. [36]. Mixtures within the biphasic region (corresponding to the mixture compositions used in the extraction experiments) were prepared by combining a specific amount of IL, salt, and water to reach a total system mass of 0.5 g. After vigorous vortex stirring and allowing the systems to equilibrate for a minimum of 12 h at (25 ± 1) °C, ATPSs were centrifuged for 5 min at 10,000 rpm (LLG-uniCFUGE5). The phases were then carefully separated and weighed, with all calculations performed as suggested by the literature [36]. For the [TBP][Sal]-based ATPS, tie-lines were determined analytically, as the equations and mass balance approaches proposed by Merchuk et al. were inadequate for describing the solubility data. Mixtures within the biphasic region were prepared by adding known amounts of [TBP][Sal], salt, and water to microtubes (total mass of 1 g), followed by vigorous vortex mixing and equilibrating for 12 h at (25 ± 1) °C. After centrifugation and phase separation, approximately 100 mg of each phase was weighed and diluted with water as needed to ensure proper detection. The content of [TBP][Sal] in the top and bottom phases was determined by UV spectroscopy using an LLG-uniSPEC2 spectrophotometer at wavelengths of 296 nm. Quantification was based on calibration curves (R^2^ > 0.9996) within the concentration range of 1–50 mg L^−1^. Water content was determined by drying each phase at 70 °C under constant stirring until a constant weight was achieved. The amount of (NH_4_)_2_SO_4_ was determined by difference based on mass balance.

### 2.3. Screening of IL-Based ATPSs for the Extraction of Pesticides

Considering the previously established liquid–liquid ternary phase diagrams, compositions of (a) 3 wt% of (NH_4_)_2_SO_4_ + 20 wt% of IL + 77 wt% of H_2_O + 5 μL of pesticide solution for [TBP][Sal] and [TBA][Sal], and (b) 23 wt% of (NH_4_)_2_SO_4_ + 20 wt% of IL + 57 wt% of H_2_O + 5 μL of pesticide solution for [TBP][Ac], [TBA][Ac], [TBP][Lac], and [TBA][Lac]-based ATPSs, were chosen for pesticide extraction. These compositions correspond to a TLL of approximately 50–60. The pesticide solution in methanol (~2.5 g/L of each compound) was used for all extraction experiments. Pre-weighted ATPS constituents (total mass of 0.5 g) were stirred and left to equilibrate for 2 h at 25 °C. After centrifugation at 6000 rpm for 5 min, the phases were separated, followed by measurement of their weight and volume. Fifty microliters of each phase were diluted with 40 vol% ethanol to a final volume of 500 µL and filtered, and the pesticide concentration was determined by ultra-performance liquid chromatography using the Waters ACQUITY UPLC system, with a PDA detector and BEH C18 column (1.7 μm, 100 mm × 2.1 mm, Waters). The mobile phase used was a mixture of water with 10 vol% acetonitrile (A) and 100 vol% acetonitrile (B). The elution gradient started at 60% (*v*/*v*) acetonitrile and was held constant for 6 min. Then, the proportion of acetonitrile was raised from 60% to 80% (*v*/*v*) over 3 min and maintained for 4 min. Subsequently, the acetonitrile proportion decreased to 60% (*v*/*v*) within 1 min and kept at this composition for an additional 1 min before the next injection. It was subsequently decreased back to 60% (*v*/*v*) within 1 min and held constant for 1 additional minute before the next injection. Detection and quantification were carried out at 220 nm for CLO and DLM and 280 nm for PYR. The elution was performed at a flow rate of 0.2 mL/min with a sample injection volume of 5 µL. The detailed experimental parameters for the UPLC method can be found in Table S1 of the Supplementary Materials. Extraction efficiencies (EE, %) of the targeted pesticides toward the IL-rich phase are calculated as

(2) $$\mathrm{EE}\;(\%)\;=\frac{{\left[\mathrm{Pest}\right]}_{\mathrm{IL}-\mathrm{rich}\;\mathrm{phase}}\cdot V_{\mathrm{IL}-\mathrm{rich}\;\mathrm{phase}}}{{\left[\mathrm{Pest}\right]}_{\mathrm{IL}-\mathrm{rich}\;\mathrm{phase}}\cdot V_{\mathrm{IL}-\mathrm{rich}\;\mathrm{phase}}+{\left[\mathrm{Pest}\right]}_{\mathrm{salt}-\mathrm{rich}\;\mathrm{phase}}\cdot V_{\mathrm{salt}-\mathrm{rich}\;\mathrm{phase}}}\;\cdot 100\;$$

where ${\left[\mathrm{Pest}\right]}_{\mathrm{IL}-\mathrm{rich}\;\mathrm{phases}}$ is the concentration of the pesticide in the IL-rich phase, ${\left[\mathrm{Pest}\right]}_{\mathrm{salt}-\mathrm{rich}\;\mathrm{phase}}$ is the concentration of pesticide in the salt-rich phase, and $V_{\mathrm{IL}-\mathrm{rich}\;\mathrm{phase}}$ and $V_{\mathrm{salt}-\mathrm{rich}\;\mathrm{phase}}$ represent the volumes of the IL- and salt-rich phases, respectively.

### 2.4. Influence of Experimental Parameters on the Extraction of Pesticides

Following the initial extraction of pesticides, the [TBP][Sal] system was selected for further in-depth investigations based on the quantities of ATPS components and the observed extraction efficiencies. These further studies focused on the influence of key parameters, such as tie-line length, mixture composition along the same tie line, temperature, and pH. To explore the effect of temperature and pH, the system consisting of 3 wt% salt and 20 wt% IL was analyzed at three different temperatures (25, 35, and 45 °C) and three pH values (3, 5, and 6). The pH values were adjusted using 1M sulfuric acid and measured with an Orion-Star A111 pH Benchtop Meter Kit at 25 ± 1 °C, with an accuracy of ±0.01 pH units. To investigate the effect of TLL on pesticide extraction efficiency, ATPS mixtures were prepared with varying concentrations of (NH_4_)_2_SO_4_ while maintaining a constant amount of [TBP][Sal]. The extraction efficiency was evaluated by varying the initial composition of the ATPS mixtures along the characterized tie line. Once the ATPS constituents were prepared (as outlined in Table 1), all systems were subjected to the procedures described in Chapter 2.3. The preconcentration factor (PF) was calculated (Equation (3)) by dividing the concentration of each pesticide in the IL-rich phase after preconcentration and ${\left[\mathrm{Pest}\right]}_{\mathrm{IL}-\mathrm{rich}\;\mathrm{phase}}$ by its initial concentration in the system, ${\left[\mathrm{Pest}\right]}_{0}$.

(3) $$\mathrm{PF}\;=\frac{{\left[\mathrm{Pest}\right]}_{\mathrm{IL}-\mathrm{rich}\;\mathrm{phase}}}{{\left[\mathrm{Pest}\right]}_{0}}$$

### 2.5. Preconcentration of Pesticides Using IL-Based ATPS

**Figure 2:**
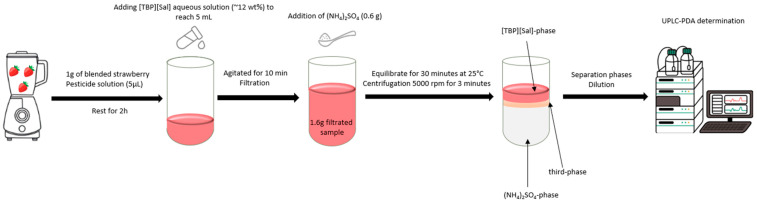
Preconcentration procedure based on IL-salt ATPS for pesticide determination in strawberry samples.

After conducting a comprehensive screening of IL-based ATPSs and evaluating various factors influencing the extraction of pesticides (CLO, PYR, and DLM), the system comprising 8 wt% [TBP][Sal] + 27 wt% (NH_4_)_2_SO_4_ + 65 wt% H_2_O at 25 °C was selected for the preconcentration of pesticides in food samples. Fresh strawberries, sourced from a local market in Belgrade, were homogenized into a uniform puree using a blender. One gram of the blended strawberry sample was spiked with 5 μL of a standard pesticide solution, with each pesticide (CLO, PYR, and DLM) at a concentration of approximately 2.5 g/L. The spiked sample was left for 2 h to ensure thorough absorption of the pesticides into the strawberry matrix, after which a 12% aqueous solution of [TBP][Sal] was added to achieve a total volume of 5 mL. This mixture was agitated on a rotary disk for 10 min and then filtered. In order to minimize salt usage, a smaller biphasic system was prepared by mixing approximately 1.6 g of the filtered sample solution (supernatant) with 0.6 g of ammonium sulfate to achieve a selected biphasic system composition. The mixture was thoroughly mixed and allowed to equilibrate for 30 min at 25 °C to promote effective phase separation. Once equilibrium was achieved, the mixture was centrifuged at 5000 rpm for 3 min to separate the phases. The salt-rich phase was carefully removed, and 50 μL was diluted with 40 vol% of ethanol to obtain a final volume of 500 µL. The IL-rich phase was further centrifuged at 15,000 rpm for an additional 2 min to ensure complete separation from any remaining salt-rich phase and interphase (third phase). Finally, 50 µL of the IL-rich phase was carefully extracted with a Hamilton syringe, followed by a dilution with 40% ethanol. After dilution, the phases were filtered through syringe filters (0.45 µm), and the samples were quantified using UPLC. Figure 2 shows the schematic steps in pesticide preconcentration.

### 2.6. Environmental and Analytical Assessment of ATPS-Based Preconcentration Using Green Metrics

The greenness assessment of the proposed ATPS-UPLC-PDA method was conducted using the AGREEprep and AGREE metrics. AGREEprep is based on 10 principles of Green Sample Preparation (GSP), while AGREE follows the 12 principles of Green Analytical Chemistry (GAC). The software for applying these metrics is freely available and cited in studies [32,33].

## 3. Results and Discussion

### 3.1. Liquid–Liquid Phase Diagrams of Phosphonium and Ammonium-Based ATPSs

The phase diagram analysis provided insight into the required quantities of each ATPS component needed to achieve two-phase separation, represented by the binodal curve, which outlines the boundaries between monophasic and biphasic regions. By comparing the binodal curves for different ionic liquids combined with the same salt, the effects of specific IL cations and anions on phase formation can be better understood. In this study, ATPSs were designed using phosphonium- and ammonium-based ILs with ammonium sulfate, a salt chosen for its strong salting-out capability and mild acidity, which aligns well with the pH of most fruit samples, promoting analyte stability and efficient partitioning.

The ternary phase diagrams of phosphonium ILs (tetrabutylphosphonium acetate, tetrabutylphosphonium lactate, and tetrabutylphosphonium salicylate) with ammonium sulfate were previously determined [34], while the phase diagrams of ammonium-based ILs (tetrabutylammonium acetate, tetrabutylammonium lactate, and tetrabutylammonium salicylate) combined with the same salt were established in this work. Experimental solubility data for each ATPS, obtained through cloud-point titration, are available in the Supplementary Materials (Table S2), alongside the parameters of the Merchuk equation (A, B, and C), their standard errors (σ), and coefficients of determination (R^2^) (Table S3). Typically, these systems show an IL-rich top phase and a salt-enriched bottom phase. However, for the [TBP][Sal]-based system, when the mixture contains 20 wt% [TBP][Sal] and 3 wt% ammonium sulfate, a phase inversion occurs, producing an IL-rich bottom phase and a salt-rich top phase. In other compositions, the system behaves conventionally, with the IL forming the upper layer and the salt-rich phase at the bottom. Due to the inability to fit experimental data from the [TBP][Sal]-based ATPS with the Merchuk equation, tie lines for these systems were determined analytically. The phase compositions at equilibrium for each extraction experiment were assessed through gravimetric or analytical methods, with results presented in Tables S4 and S5 of the Supplementary Materials.

**Figure 3:**
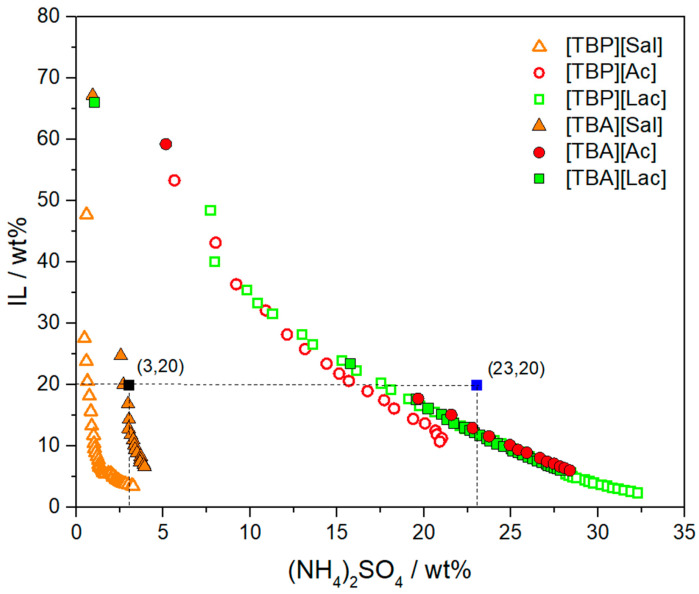
Ternary phase diagrams composed of [TBP]-IL + (NH_4_)_2_SO_4_ + H_2_O at 25 °C and *p* = 0.1 MPa (open symbols) [34] and [TBA]-IL + (NH_4_)_2_SO_4_ + H_2_O at 25 °C and *p* = 0.1 MPa (full symbols). Compositions of ATPS mixture selected for TLs and extraction study (■, [TBP][Sal] or [TBA][Sal]-based ATPS, and ■, [TBP][Ac] or [TBA][Ac] or [TBP][Lac] or [TBA][Lac]-based ATPS).

All tested phosphonium- and ammonium-based ILs underwent liquid–liquid demixing in the presence of ammonium sulfate. Despite the overlapping same binodal curves (Figure 3), the efficacy of these ILs in forming ATPSs at 20 wt% (the concentration used in extraction studies) follows the order: [TBP][Sal] > [TBA][Sal] > [TBP][Ac] > [TBP][Lac] > [TBA][Lac] > [TBA][Ac]. Among these, [TBP][Sal] shows the highest capacity to promote two-phase formation with ammonium sulfate, while [TBA][Ac] demonstrates the lowest. Systems with [TBP][Sal] and [TBA][Sal] displayed substantial biphasic regions with binodal curves close to the origin; however, [TBP][Sal] required significantly lower concentrations to induce ATPS formation than [TBA][Sal]. The same trend was observed when lactate or acetate anions were used instead of salicylate, with ILs of the same anion following the order: [TBP] > [TBA]. This behavior is due to the higher hydrophobicity of the phosphonium cation, which promotes phase separation more effectively than the ammonium cation [37]. The same behavior of [TBP][Sal] and [TBA][Sal] was observed when using citrate salt as the salting-out agent [28].

The effect of anions on ATPS formation was assessed using ILs with the same cation but different anions. For ILs with the [TBP] cation, the ability to form ATPSs decreased in the following order: [Sal] > [Ac] > [Lac], aligning with their octanol-water partition coefficients (logK_ow_) of 1.977, −0.223, and −0.472, respectively [38]. For ammonium ILs, the trend in efficiency followed [Sal] > [Lac] > [Ac]. This discrepancy can be attributed to the stronger cation–anion interactions in TBA-based ILs, as confirmed by the solid state of [TBA][Lac] at room temperature, in contrast to the liquid state of [TBP][Lac]. Previously, it has been shown that ion-pair binding energy between the cation and anion in ILs correlates well with melting point [39]. The higher degree of molecular packing and binding energy in [TBA][Lac] reduces interactions with water and enhances phase-separation tendencies in ATPSs. Notably, [TBP][Sal] formed an ATPS more easily than other investigated ILs, requiring exceptionally low sulfate concentrations (<3%) to induce phase separation. This underscores the importance of the salicylate anion hydrophobicity, as its high logK_ow_ value facilitates the formation of two-phase systems.

### 3.2. Screening of IL-Based ATPSs for the Extraction of Pesticides

**Figure 4:**
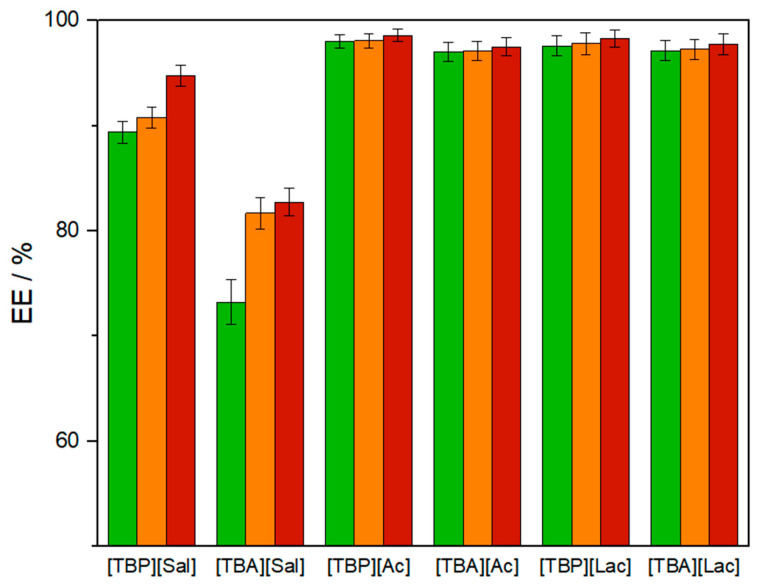
Extraction efficiencies of CLO (green bar), PYR (orange bar) and DLM (red bar) in ATPSs composed of: 3 wt% of (NH_4_)_2_SO_4_ + 20 wt% of IL + 77 wt% of H_2_O for [TBP][Sal] and [TBA][Sal], and 23 wt% of (NH_4_)_2_SO_4_ + 20 wt% of IL + 57 wt% of H_2_O for [TBP][Ac], [TBA][Ac], [TBP][Lac], and [TBA][Lac] at 25 °C and *p* = 0.1 MPa.

The extraction capabilities of phosphonium and ammonium-based ATPSs for three pesticides (CLO, PYR, and DLM) from aqueous solutions were evaluated. Optimal compositions of ternary ATPS mixtures (illustrated in Figure 3) were selected as follows: (a) 3 wt% of (NH_4_)_2_SO_4_ + 20 wt% of IL + 77 wt% of H_2_O + 5 μL of pesticide solution for [TBP][Sal] and [TBA][Sal], and (b) 23 wt% of (NH_4_)_2_SO_4_ + 20 wt% of IL + 57 wt% of H_2_O + 5 μL of pesticide solution for [TBP][Ac], [TBA][Ac], [TBP][Lac], and [TBA][Lac]-based ATPSs. To maintain a consistent comparison, a tie line length of 50–60 with a constant wt% of IL was targeted for each mixture. The extraction efficiencies for CLO, PYR, and DLM with respective standard deviations are shown in Figure 4. At the pH values of the ATPS mixtures (6–7), all pesticides are present in neutral forms, meaning that their hydrophobicity largely determines their partitioning behavior, as reflected in the extraction efficiency trend (EE_CLO_ < EE_PYR_ < EE_DLM_), aligning with the pesticides logK_ow_ values (2.93 < 4.70 < 5.74) [38].

The highest EE for each pesticide was observed in the (NH_4_)_2_SO_4_/[TBP][Ac] ATPS: 98.01 ± 0.66% for CLO, 98.07 ± 0.63% for PYR, and 98.59 ± 0.59% for DLM. In contrast, the (NH_4_)_2_SO_4_/[TBA][Sal] ATPS had slightly lower efficiency (EE_CLO_ = 73.23 ± 2.09%, EE_PYR_ = 81.7 ± 1.5%, and EE_DLM_ = 82.75 ± 1.33%). However, the trend in EE for each pesticide across ATPS compositions—[TBP][Ac] > [TBP][Lac] > [TBA][Lac] > [TBA][Ac] > [TBP][Sal] > [TBA][Sal]—did not fully follow the order of ATPS formation. This discrepancy can be attributed to differing tie-line lengths; systems with longer TLs tend to have higher EEs and preconcentration factors, along with lower cross-contamination between phases. For instance, in systems with long TLs, the IL-rich phase contains minimal sulfates, while the salt-rich phase primarily consists of water and salt. The [TBA][Sal]-ATPS system exhibits lower extraction efficiency, with a TLL of 32, while others range from 50 to 60. Table S4 illustrates that the [TBP][Ac]/(NH_4_)_2_SO_4_ system contains less than 1% IL in the salt-rich phase, resulting in the highest extraction efficiency across all pesticides. Conversely, the [TBA][Sal]-based ATPS has the lowest extraction efficiency, with over 12% IL in the salt-rich phase. A high concentration of IL in the salt-rich phase can reduce the salting-out effect, weakening the ionic strength and decreasing the phase’s ability to exclude analytes. Considering the component quantities, phase volume ratios, and extraction efficiencies, the [TBP][Sal]/ammonium sulfate ATPS presents itself as a promising extraction medium for CLO, PYR, and DLM from food matrices. Furthermore, [TBP][Sal] exhibits low cytotoxicity [28] with minimal loss or cross-contamination into the salt-rich phase.

### 3.3. Optimization of Experimental Parameters for Extraction of Pesticides

After conducting the initial screening of ATPSs for extracting clomazone, pyraclostrobin, and deltamethrin, the results revealed that the most effective candidate for extracting all three pesticides is the [TBP][Ac] + (NH_4_)_2_SO_4_ + H_2_O system, exhibiting a high extraction efficiency exceeding 98%. This efficiency was achieved using a system comprising 20 wt% IL and 23 wt% salt. Conversely, utilizing 20 wt% [TBP][Sal] with significantly lower salt content (3 wt%) yielded slightly lower efficiencies ranging from 90 to 94%. Given the minimal differences in extraction efficiencies among these systems and the substantial variation in required constituent amounts, the [TBP][Sal]/(NH_4_)_2_SO_4_ system was chosen for further investigation. Several operational parameters, including temperature, pH, TLL, and mixture composition along the same TL (phase ratio), were investigated for their effect on the extraction of pesticides. This approach enables the identification of optimal conditions for the extraction and preconcentration process while utilizing fewer components, thereby reducing costs, energy consumption, and environmental impact.

**Figure 5:**
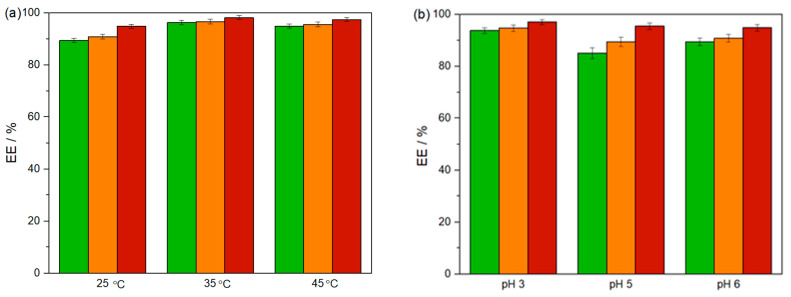
Extraction efficiencies of CLO (green bar), PYR (orange bar) and DLM (red bar) in ATPSs at *p* = 0.1 MPa composed of 3 wt% of (NH_4_)_2_SO_4_ + 20 wt% of [TBP][Sal] + 77 wt% of H_2_O (**a**) on 25, 35 and 45 °C and (**b**) on 25 °C at pH 3, pH 5, and pH 6.

Temperature and pH influence. The influence of the temperature (25, 35, and 45 °C) on EE values of CLO, PYR, and DLM are shown in Figure 5a. At 35 °C, extraction efficiencies for all three pesticides increase by approximately 7% compared to those at 25 °C, while a smaller increase of 5% is observed at 45 °C. This suggests a slightly positive effect of temperature on extraction efficiency. Given the slight dependence of EEs on temperature changes (89.39 ± 0.80% > EE_CLO_ > 96.22 ± 0.82%, 90.79 ± 0.95% > EE_PYR_ > 96.62 ± 0.95% and 94.77 ± 0.79% > EE_DLM_ > 98.14 ± 0.79%) and aiming to minimize energy and the operational costs of the process, the room temperature, i.e., 25 °C, can be taken as optimal.

The pH effect on extraction pesticides was examined for systems without pH adjustment (pH ≈ 6) and for systems adjusted to pH ≈ 5 and ≈3 using 1M sulfuric acid. This pH range aligns with the typical acidity levels found in most fruit juices, such as strawberries, which have a pH of 3.54 ± 0.01. PYR and DLM are in neutral form in the whole pH working range, while clomazone is neutral at pH > 4 and at pH = 3 is 50% negatively charged [38]. However, the results (85.01 ± 2.13% > EE_CLO_ > 93.69 ± 1.06%, 89.33 ± 1.78% > EE_PYR_ > 94.66 ± 1.25%, and 95.58 ± 1.20% > EE_DLM_ > 97.00 ± 0.96%) indicate that pH has a small effect on EE parameter of each compound (Figure 5b). Based on the minimal influence of pH on extraction efficiency observed in this study, future experiments will use an unadjusted ATPS without acid addition. This approach reduces the number of components involved and eliminates the need for acidic modifiers, making the process more environmentally friendly and aligned with green chemistry principles. Eliminating pH adjustments in the extraction process enhances sustainability and simplifies the method, making it more feasible for practical applications.

TLL and phase ratio influence. The system formed by [TBP][Sal] and (NH_4_)_2_SO_4_, as mentioned before, exhibits a wide biphasic region, resulting in a long tie-line length. A long TLL reduces cross-contamination between phases enriched with opposite components (the IL phase contains very little ammonium salt, while the salt phase contains only salt and water). Adjusting the mixture compositions along the same tie line allows for the tailoring of the volumes of the coexisting phases without altering their composition and enables higher preconcentration factors [40].

**Figure 6:**
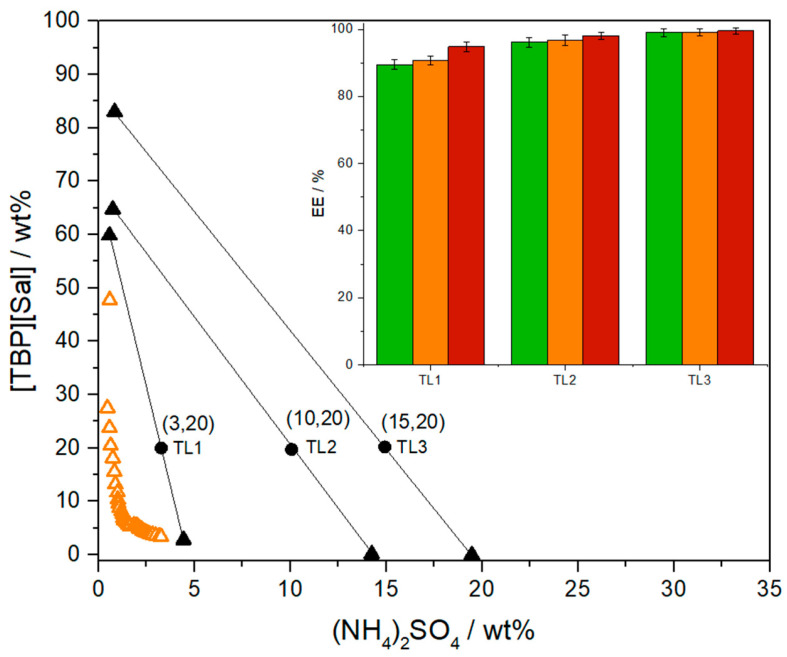
Effect of different TLs on extraction efficiencies of CLO (green bar), PYR (orange bar), and DLM (red bar) in ATPSs composed of (NH_4_)_2_SO_4_ + [TBP][Sal] + H_2_O on 25 °C at 25 °C and *p* = 0.1 MPa. Binodal curve data (△), TL (▲), and mixture compositions (●).

The impact of TLL on pesticide extraction was examined by varying the concentrations of (NH_4_)_2_SO_4_ (3.28, 10.07, and 14.88 wt%) while maintaining [TBP][Sal] at ~20 wt%. Figure 6 demonstrates how the extraction of CLO, PYR, and DLM is influenced by increasing TLL values: 57.17, 65.94, and 66.96. By increasing the TLL under selected conditions (pH ≈ 6 and T = 25 °C), the extraction efficiencies increase from 89.39 ± 1.45% to 98.94 ± 1.26% for CLO, from 90.79 ± 1.33% to 99.12 ± 1.04% for PYR, and from 94.77 ± 1.55% to 99.48 ± 0.98% for DLM. Given that the longest TLL (TL3, ~67) resulted in over 98% extraction efficiency for all tested pesticides and only 0.01% ionic liquid remained in the salt-rich phase (Table S5), this condition was selected for further investigation.

**Figure 7:**
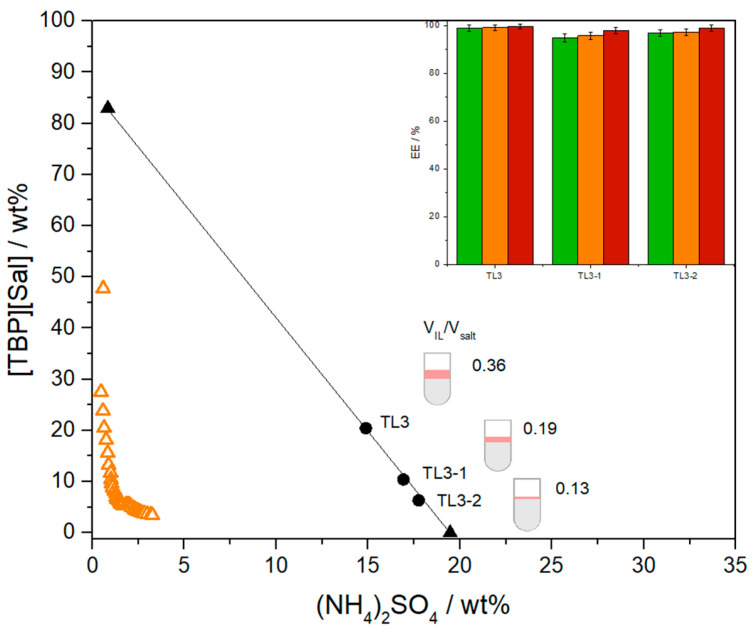
Effect of different phase ratio (VIL/Vsalt) on extraction efficiencies of CLO (green bar), PYR (orange bar), and DLM (red bar) in ATPSs composed of (NH4)_2_SO_4_ + [TBP][Sal] + H_2_O on 25 °C at 25 °C and *p* = 0.1 MPa. Binodal curve data (△), TL (▲), and mixture compositions (●).

Several systems with varying V_IL-phase_/V_salt-phase_ ratios were investigated along TL3 (~67 in length), and the obtained extraction efficiencies for pesticides are shown in Figure 7. The EEs decreased from 98.94 ± 1.26% to 94.91 ± 1.66% for CLO, from 99.12 ± 1.04% to 95.1.60 ± 1.40% for PYR and from 99.48 ± 0.98% to 97.8 ± 1.4 for DLM, as ratios of phases decline from 0.36 to 0.13. Preconcentration factors of 3.3- to 11.5-fold for CLO, 3.7- to 12.3-fold for PYR, and 3.0- to 14.6-fold for DLM were achieved, demonstrating the potential to concentrate pesticides in the IL-rich phase by up to 14-fold with only a 4% decrease in extraction efficiency. These initial findings suggest that the [TBP][Sal]-based ATPS offers a promising method for pesticide preconcentration, facilitating their detection and quantification by UPLC.

### 3.4. Preconcentration of Pesticides Using IL-Based ATPSs

**Figure 8:**
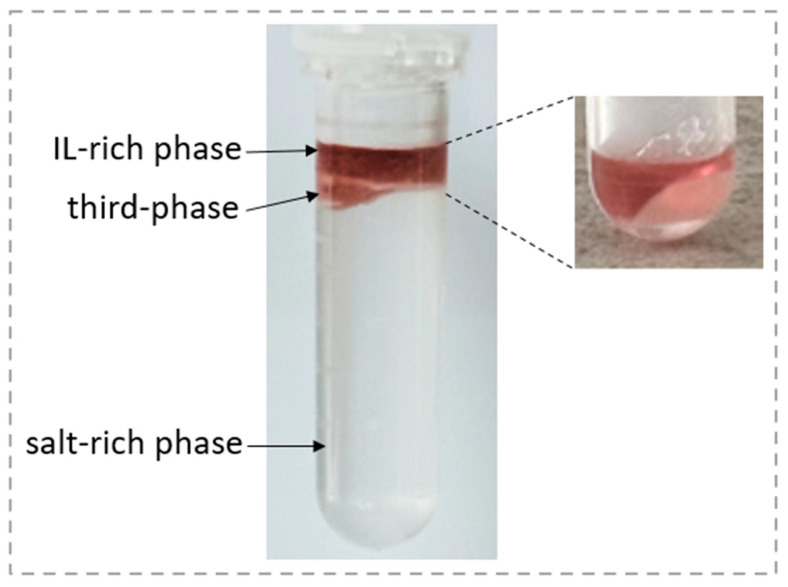
A photograph of the [TBP][Sal]/(NH_4_)_2_SO_4_ ATPS used for pesticide preconcentration in a strawberry sample, showing three distinct phases: IL-rich phase, third-phase, and salt-rich phase. The inset highlights the IL-rich phase and the third phase after the salt-rich phase has been removed.

Pesticide monitoring, particularly in food samples where concentrations often fall below the MRLs, places greater importance on preconcentration than on extraction efficiency. Achieving adequate preconcentration is essential for detecting even trace levels of pesticides, as analytical devices may struggle to identify them at such low concentrations without a significant preconcentration step [16]. By increasing the preconcentration factor, achieved through maximizing the ratio of pesticide concentration in the IL-rich phase to its initial concentration, the method significantly enhances the preconcentration of pesticides. This is largely due to the substantial volume difference between the IL-rich phase and the original sample volume, allowing trace amounts of pesticides to be more readily detected and quantified. Higher volume ratios are attained by selecting a biphasic system composition located deep within the biphasic region [24,26]. This composition requires minimal ionic liquid for phase formation but results in a higher salt concentration. As a result, a composition of 8% IL, 27% salt, and 65% water, with a volume ratio of 0.07 (0.125 mL/1.800 mL) at 25 °C, was chosen for the preconcentration of pesticides from the real sample. Initially, 12 wt% aqueous solution of [TBP][Sal] was introduced to the spiked strawberry, followed by agitation for 10 min to promote interaction with the target compounds. The mixture was then filtered, and ammonium sulfate was added to the supernatant to induce ATPS formation at 25 °C. Once equilibrium was reached, the mixture was centrifuged, resulting in three distinct phases: a top IL-rich phase, a bottom salt-rich phase, and a third phase (TP) (Figure 8).

The third phase only appeared after the addition of salt, whereas a supernatant without salt did not form an interphase. This suggests that components from the strawberry matrix, such as fibers and carbohydrates, were salted out, leading to the formation of the third phase. Despite the presence of the third phase, it did not interfere with the experimental sampling, as only 50 μL of phases were required for analysis. Initially, the entire salt-rich bottom phase was withdrawn using a syringe, followed by further centrifugation of the top IL-rich phase along with the interphase layer. After this additional step, 50 μL of the top IL-rich phase was collected, diluted, and analyzed using UPLC to quantify pesticide residue. The UPLC analysis showed that pesticides were extracted into the IL-rich phase, with extraction efficiencies of 72.20 ± 1.98% for CLO, 74.24 ± 1.89% for PYR, and 82.40 ± 1.56% for DLM. The slightly lower extraction efficiencies can be attributed to the complexity of the real sample, as well as the existence of the third phase, which likely retained some pesticide residues.

**Figure 9:**
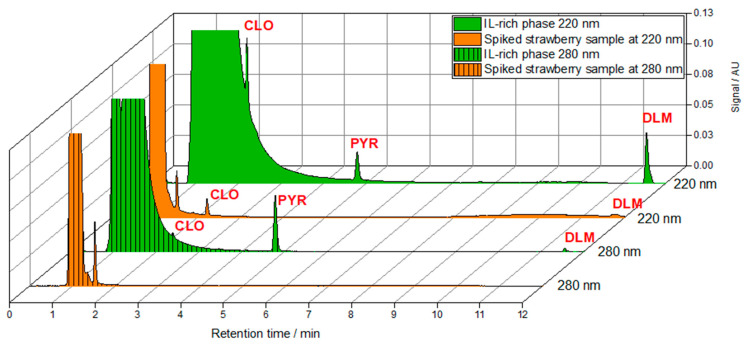
Chromatograms at 220 nm and 280 nm of the spiked strawberry sample with clomazone (CLO), pyraclostrobin (PYR), and deltamethrin (DLM) without ATPS pre-treatment and chromatograms at 220 nm and 280 nm of the IL-rich phase with clomazone (CLO), pyraclostrobin (PYR) and deltamethrin (DLM) after preconcentration with ATPSs.

Figure 9 presents chromatograms at 220 nm and 280 nm of the spiked strawberry sample, both without ATPS pre-treatment and after preconcentration with ATPS. In the post-preconcentration chromatogram, the pesticide peaks are clearly visible and quantifiable, demonstrating a 16-fold preconcentration in a single-step process. Given that the LOD values for UPLC are 0.1 mg/L for CLO and PYR and 0.05 mg/L for DLM (Table S1), this method enables the quantification of pesticide-contaminated samples at concentrations around the μg/L range. Considering the established MRLs for strawberries (CLO 0.01 mg/L, PYR 1.5 mg/L, and DLM 0.2 mg/L) [41], the proposed aqueous two-phase extraction method, using tetrabutylphosphonium salicylate, proves effective for monitoring pesticide residues in strawberries. The analysis of the chromatograms reveals distinct peaks for each pesticide without any interference from other compounds found in strawberries, indicating that both cleaning and preconcentration were effectively accomplished in a single step. This clarity in the chromatographic data facilitates accurate quantification of clomazone, pyraclostrobin, and deltamethrin individually. The findings underscore the efficacy of the developed one-step clean-up and preconcentration method utilizing the [TBP][Sal]-based aqueous two-phase system. Additionally, the IL-ATPS approach could be expanded to target a broader range of pesticide classes, including those with varying polarities or solubilities. By adjusting ionic liquid or salt combinations, the system can be tailored to selectively extract diverse pesticides, enhancing its application for comprehensive food safety monitoring. In addition to phosphonium- and ammonium-based ionic liquids, cholinium-based ionic liquids present a compelling alternative due to their low toxicity and biodegradability. Their use could further improve the method’s green chemistry profile and broaden its validation for other pesticides and matrices. The flexibility and adaptability of this method, combined with the exploration of different ionic liquid types, establish it as a promising solution for addressing food safety challenges across diverse and complex matrices. Future research should prioritize testing various types of ionic liquids and optimizing the system for more challenging matrices, such as grains, high-fiber foods, and root vegetables, to fully validate its robustness and expand its applicability.

### 3.5. Environmental and Analytical Assessment of IL-Based ATPSs for Preconcentration of Pesticides Using Green Metrics

The proposed method for preconcentrating pesticides using aqueous two-phase systems with ionic liquids aligns with several principles of green chemistry, such as eliminating the need for derivatization, minimizing analytical waste, and utilizing low-toxicity reagents like ionic liquids [42]. Notably, Jocić et al. [28] demonstrated that [TBP][Sal] concentrations as low as 0.01% in the bottom aqueous phase exhibited minimal cytotoxicity, showing no significant adverse effects on MRC-5 cells. This reinforces the environmental and safety advantages of using [TBP][Sal] in pesticide preconcentration methods.

To assess the environmental sustainability of the proposed ATPS-UPLC-PDA method, both the AGREEprep and AGREE metrics were applied. AGREEprep focuses specifically on sample preparation, evaluating compliance with the 10 Green Sample Preparation principles, while AGREE examines the entire analytical workflow through the 12 Green Analytical Chemistry principles. The evaluations use a color-coded system (green for high compliance to red for low) and provide an overall score between 0 and 1, where a higher score indicates better environmental performance [32,33].

**Figure 10:**
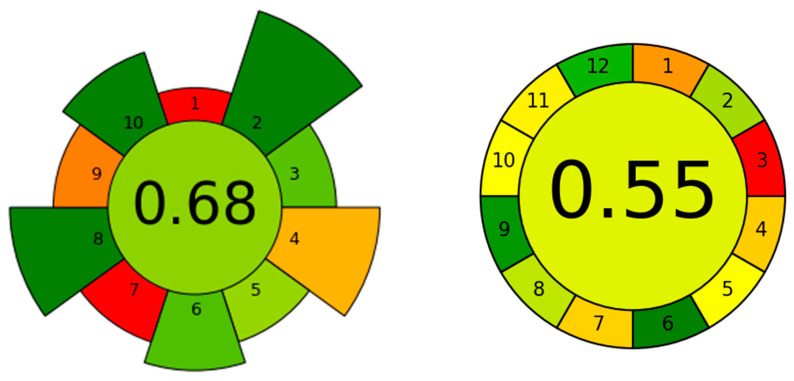
The results of AGREEprep and AGREE assessment of procedure for preconcentration of pesticides using [TBP][Sal]/(NH_4_)_2_SO_4_ ATPS without UPLC (**left**) and with UPLC (**right**).

In this study, as shown in Figure 10, AGREEprep scored 0.68, with most parameters aligning well with Green Analytical Chemistry principles. According to AGREEprep, the greenest features of the proposed IL-ATPS method are the use of non-hazardous materials (criterium 2), as this method involves only a low-cytotoxicity IL and a safe inorganic salt as reagents, contributing to a green rating for operator safety (criterium 10). Additionally, low energy consumption (criterium 8) is achieved using a disk rotator, vortex, and centrifugation, requiring just 10 Wh of energy input. Less eco-friendly aspects include the 1 g sample size (criterium 5) and a throughput of 25 samples per hour (criterium 6). However, a few areas—criterium 1 (ex situ preparation), criterium 4 (5.6 g waste), criterium 7 (manual preparation with six steps or fewer), and criterium 9 (UPLC use)—were flagged as less eco-friendly. While the AGREEprep evaluates only the sample preparation process, AGREE extends its analysis to include the entire workflow, covering both sample preparation and detection. With an AGREE score of 0.55, the method demonstrates notable green chemistry features, such as 1 g of sample (criterium 2), the absence of a derivatization step (criterium 6), and analysis of three analytes in a single run with a throughput of five samples per hour (criterium 8). The UPLC, consuming only 0.217 kWh (criterium 9), contributes to its eco-friendly profile, alongside operator safety (criterium 12), with highly flammable acetonitrile being the only hazardous solvent used. However, some aspects of the ATPS-UPLC-PDA method are less green: manual preparation of miniaturized samples (criterium 5), the use of bio-based reagents (criterium 10), and the small quantities of acetonitrile (1.2 mL) in the mobile phase (criterium 11). Additionally, the method’s less green features include six or more distinct steps in sample preparation (criterium 4) and the generation of 8.6 g of waste (criterium 7). Similar to the AGREEprep method, the least green steps are the multi-step sample pre-treatment (criterium 1) and off-line measurement (criterium 3). The UPLC instrumentation, selected for its low energy requirements and minimized use of ACN, contributes significantly to the method’s eco-friendliness. Furthermore, UPLC generally requires smaller volumes of mobile phase compared to HPLC, making it a greener choice in this context. This reduced solvent usage aligns well with sustainable practices, even though the method includes off-line measurements. Additionally, as previously noted, ionic liquid-based samples are particularly suited to liquid chromatography due to their low melting points, which prevent volatility issues common in gas chromatography. These factors together strengthen the environmental profile of the method by balancing resource efficiency with practical analytical performance.

For comparison, Figure S4 in Supplementary Materials displays the AGREEprep and AGREE assessment diagrams applied to a study reported in the literature [9]. A QuEChERS-based sample preparation method combined with ultra-performance liquid chromatography–tandem mass spectrometry (UPLC-MS/MS) was established for the determination of 43 pesticide residues in strawberries. Optimized clean-up sorbents of 50 mg PSA, 50 mg C18, and 150 mg MgSO₄ demonstrated the best performance in dispersive solid-phase extraction (d-SPE). The proposed ATPS-UPLC-PDA method in this study achieved higher green scores in both AGREE and AGREEprep compared to the QuEChERS-UPLC-MS/MS method, scoring 0.68 and 0.55, respectively, while the method from the literature scored only 0.35 and 0.3. These differences are primarily due to the QuEChERS method’s reliance on hazardous chemicals, such as 10 mL of highly flammable organic solvents (ACN) as extractant, a larger sample size, and consequently more waste generated. The findings indicate that IL-ATPS provides a greener alternative for pesticide preconcentration, with minimized solvent use, reduced waste, and alignment with sustainable analytical practices.

## 4. Conclusions

This study introduces a greener method for monitoring pesticides in food samples, focusing on strawberries, by addressing the challenge of low pesticide concentrations through an ionic liquid-based aqueous two-phase system (IL-ATPS) for preconcentration and UPLC-PDA detection. To achieve this, phase diagrams and initial extraction studies were conducted for clomazone, pyraclostrobin, and deltamethrin using phosphonium- and ammonium-ionic-based IL-ATPS with ammonium sulfate. Extraction efficiencies exceeded 90%, except for tetrabutylammonium salicylate-based ATPS, which yielded 73%. Among the tested ILs, the most hydrophobic—tetrabutylphosphonium salicylate—was the most efficiently salted out, achieving over 98% extraction efficiency for each pesticide after optimizing parameters such as temperature, pH, tie-line length, and system composition. The developed ATPS technology was successfully applied to strawberry samples, achieving preconcentration factors of up to 16-fold, reaching mg/L levels that allow for detection using UPLC-PDA.

The environmental assessments conducted with AGREEprep and AGREE tools confirmed the alignment of the ATPS-UPLC-PDA method for pesticide determination in strawberries with green chemistry principles. The AGREEprep score of 0.68 and AGREE score of 0.55 underscore the method’s sustainable aspects, including low solvent use, minimized waste, the use of a low-cytotoxicity ionic liquid, and minimal use of hazardous reagents like acetonitrile. The inclusion of UPLC, selected for its low energy consumption and minimal ACN use, enhances the overall environmental profile in the AGREE evaluation, emphasizing the sustainability of both the sample preparation and detection stages. Overall, this IL-ATPS platform not only achieves efficient pesticide preconcentration from strawberries but also holds promise for broader application with reduced environmental impact. Future work will explore expanding this method to other pesticide types and matrices while further optimizing its green profile.

## Figures and Tables

**Table 1:** Selected compositions (wt%), temperature (°C), and pH for evaluating pesticide extraction in ATPSs: (NH_4_)_2_SO_4_ (X) + [TBP][Sal] (Y) + H_2_O (Z) + 5 μL pesticide solution.

	100·X	100·Y	100·Z	T	pH
Temperature influence					
T25	3	20	77	25	6
T35	3	20	77	35	6
T40	3	20	77	45	6
pH influence					
pH3	3	20	77	25	3
pH5	3	20	77	25	5
pH6	3	20	77	25	6
Tie-line length influence					
TL1	3	20	77	25	6
TL2	10	20	70	25	6
TL3	15	20	65	25	6
Phase ratio influence					
TL3	15	20	65	25	6
TL3b	17	10	73	25	6
TL3c	18	5	77	25	6

## Data Availability

The original contributions presented in this study are included in the article/Supplementary Materials. Further inquiries can be directed to the corresponding author.

## Supplementary Materials

The following supporting information can be downloaded at https://www.mdpi.com/article/10.3390/foods13244106/s1, Figures S1–S3: FTIR spectra of the synthesized ionic liquids; Figure S4: AGREEprep and AGREE assessment for QuEChERS-UPLC-MS/MS; Table S1: Parameters for the UPLC-PDA analytical method; Table S2: Experimental binodal mass fraction data for the salt (X) + IL (Y) + H_2_O ATPS at 25 °C and at *p* = 0.1 MPa.; Table S3: Correlation parameters, standard deviations and determination coefficients (R2) of the salt (X) + IL (Y) + H_2_O ATPS obtained by the Merchuk equation at 25 °C and at *p* = 0.1 MPa); Table S4: Experimental tie-lines data in percentage weight fraction for the ATPS composed of (salt (X) + IL (Y) + H_2_O (Z)) at 25 °C, and volume ratios (Vr).; Table S5: Experimental tie-lines data in percentage weight fraction for the ATPS composed of (salt (X) + [TBP][Sal] (Y) + H_2_O (Z)) at 25 °C, and volume ratios (Vr).
