# Supplementary material for: Green Pre-Treatment Strategy Using Ionic Liquid-Based Aqueous Two-Phase Systems for Pesticide Determination in Strawberry Samples
Source: Foods. 2024 Dec 18;13(24):4106. doi: 10.3390/foods13244106 (PMC11675850; doi:10.3390/foods13244106)
Supplement: Supplementary file 1 [file foods-13-04106-s001.zip › foods-3379256-supplementary.pdf]

Supplementary Materials

Green Pre-Treatment Strategy Using Ionic Liquid-Based Aqueous Two-Phase Systems for Pesticide Determination in Strawberry Samples

Ana Jocić [1], Slađana Marić [1,*], Danijela Tekić [1], Jasmina Mušović [1], Jelena Milićević [2], Sanja Živković [1] and Aleksandra Dimitrijević [1]

[1] Department of Physical Chemistry, VINČA Institute of Nuclear Sciences—National Institute of the Republic of Serbia, University of Belgrade, Mike Petrovića Alasa 12–14, 11351 Belgrade, Serbia; ana.jocic@vin.bg.ac.rs (A.J.); danijela.tekic@vin.bg.ac.rs (D.T.); jasmina.musovic@vin.bg.ac.rs (J.M.); sanjaz@vin.bg.ac.rs (S.Ž.); daleksandra@vin.bg.ac.rs (A.D.)

[2] Department for Bioinformatics and Computational Chemistry, VINČA Institute of Nuclear Sciences—National Institute of the Republic of Serbia, University of Belgrade, Mike Petrovića Alasa 12–14, 11351 Belgrade, Serbia; jdjordjevic@vin.bg.ac.rs

* Correspondence: sladjana.maric@vin.bg.ac.rs

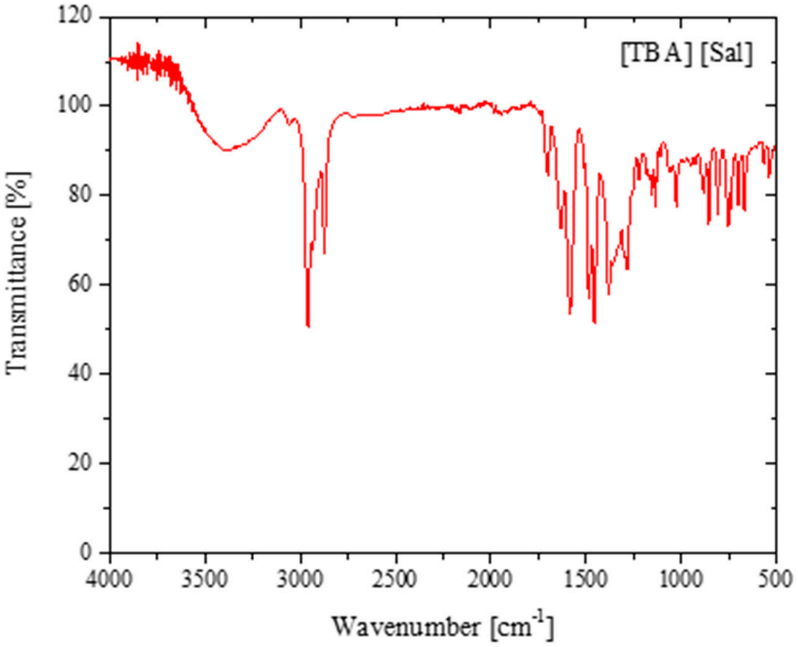

Figure S1. FTIR spectra of the synthesized [TBA][Sal].

IR (neat): 1025 (TBA stretching C-N); 1284 (Salycilate stretching C-C); 1456 (Salycilate stretching C=C); 1584 Salycilate C=C stretching); 2873 (TBA stretching C-H); 2959 (TBA stretching C-H)

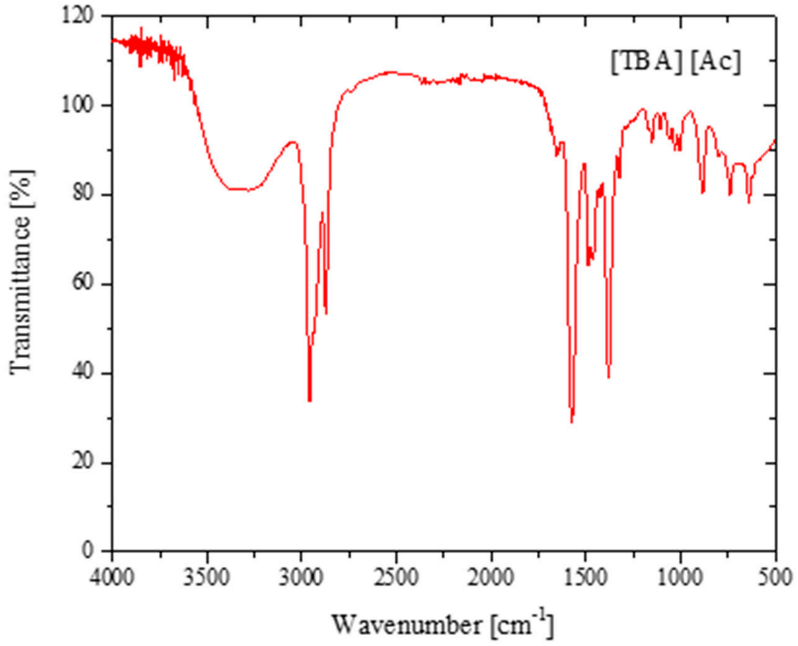

Figure S2. FTIR spectra of the synthesized [TBA][Ac].

IR (neat): 3281 (stretching O-H); 2959-2874 (sym. stretching C-H, (CH_3)); 1575 (sym. stretching COO^-); 1488-1379 (bending C-H (CH_3)); 1323 (sym. stretching CH_3); 1151 (stretching C-CH_3); 1004 (asym. stretching C-C-O); 1025 (TBA stretching C-N).

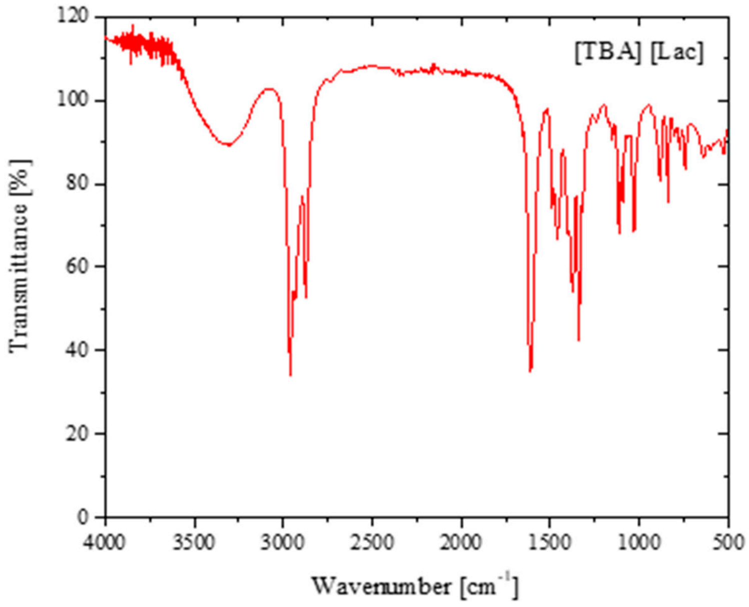

Figure S3. FTIR spectra of the synthesized [TBA][Lac].

IR (neat): 3302 (stretching O-H); 2960-2874 (sym. stretching C-H, (CH_3)); 1611 (sym. stretching COO^-); 1489-1373 (bending C-H (CH_3)); 1092 (stretching C-CH_3); 1029 (stretching CH_2-OH); 1029 (TBA stretching C-N);

Table S1. Parameters for the UPLC-PDA analytical method.

	**Clomazone**	**Pyraclostrobin**	**Deltamethrin**
**Retention Time** (min)	2.17	4.72	11.54
λ (nm)	220	280	220
**Number of Standards**	6	6	6
**Linearity Range** (mg/L)	0.2-12	0.1-12	0.1-12
R^2	0.9997	0.9994	0.9980
**LOD** *(taken as signal/noise > 3)*	0.10	0.10	0.05
**LOQ** *(taken as signal/noise > 10)*	0.25	0.25	0.10

Table S2. Experimental binodal mass fraction data for the $(NH_4)_2SO_4$ (X) + IL (Y) + H_2O ATPS at 25°C and at p = 0.1 MPa.

**[TBP][Sal]** [1]		**[TBP]Ac** [1]		**[TBP][Lac]**[1]		**[TBA][Sal]**		**[TBA][Ac]**		**[TBA][Lac]**	
100·X	100·Y	100·X	100·Y	100·X	100·Y	100·X	100·Y	100·X	100·Y	100·X	100·Y
0.39	47.74	5.64	53.31	7.72	48.44	0.94	67.18	5.15	59.23	1.03	66.15
0.47	27.54	8.01	43.12	7.97	40.03	24.69	2.56	19.67	17.69	15.74	23.46
0.57	23.80	9.20	36.34	9.80	35.39	20.00	2.71	21.57	15.06	19.53	17.64
0.64	20.48	10.89	32.09	10.43	33.31	16.80	2.97	22.79	12.91	20.21	16.14
0.75	18.13	12.11	28.14	11.28	31.48	14.29	3.03	23.73	11.54	20.97	15.17
0.84	15.60	13.15	25.80	12.99	28.14	12.76	3.00	24.93	10.13	21.30	14.34
0.88	13.24	14.40	23.39	13.60	26.52	11.80	3.13	25.42	9.36	21.67	13.74
0.99	11.73	15.12	21.76	15.27	23.91	10.95	3.28	25.91	8.87	22.29	13.01
1.01	10.39	15.68	20.59	16.12	22.25	10.12	3.34	26.67	8.01	22.60	12.57
1.05	9.59	16.75	18.93	17.53	20.19	9.39	3.38	27.08	7.43	22.87	12.17
1.10	8.85	17.70	17.44	18.11	19.11	8.81	3.50	27.47	7.09	23.18	11.75
1.20	8.23	18.28	16.08	19.12	17.63	8.26	3.71	27.83	6.60	23.60	11.25
1.28	7.71	19.37	14.37	19.75	16.52	7.77	3.70	28.07	6.37	23.78	10.85
1.30	6.94	20.05	13.64	20.60	15.50	7.29	3.67	28.39	5.97	24.18	10.40
1.33	6.54	20.65	12.46	21.44	14.54	6.94	3.88			24.54	9.92
1.44	6.28	20.73	11.83	21.70	13.76	6.59	3.93			24.98	9.53
1.51	5.93	21.02	11.22	22.09	13.26					25.10	9.23
1.56	5.64	20.90	10.68	22.39	12.72					25.37	8.89
1.66	5.38			23.73	11.38					25.66	8.55
1.85	5.53			24.02	10.89					25.95	8.27
1.99	5.51			24.42	10.36					26.22	7.91
2.10	5.18			24.83	9.89					26.49	7.61
2.12	4.95			25.05	9.54					26.74	7.35
2.25	4.76			25.53	8.97					27.04	7.06
2.31	4.54			25.66	8.69					27.13	6.83
2.40	4.36			26.07	8.25					27.29	6.65
2.49	4.19			26.45	7.83					27.45	6.49
2.61	4.02			26.66	7.55					27.65	6.29

2.69	3.88			26.83	7.31					27.84	6.07
2.82	3.78			27.10	7.02						
2.98	3.65			27.47	6.57						
3.19	3.53			27.76	6.31						
3.27	3.40			27.96	6.08						
				28.14	5.87						
				28.43	5.64						
				28.15	5.39						
				28.32	5.15						
				28.55	4.95						
				28.77	4.77						
				29.23	4.45						
				29.49	4.18						
				29.74	3.93						
				30.19	3.65						
				30.51	3.41						
				30.82	3.15						
				31.16	2.97						
				31.51	2.72						
				31.89	2.52						
				32.28	2.28						

[1]The standard uncertainty of the measured percentage weight fraction is $u(100 \cdot y) = u(100 \cdot x) = 0.052$, $u(T) = 1$ ºC. Relative standard uncertainty: $u_r\,(p) = 1.5\%$

Table S3. Correlation parameters (A, B and C), standard deviations (σ) and determination coefficients (R^2) of the $(NH_4)_2SO_4$ (X) + IL (Y) + H_2O ATPS obtained by the Merchuk equation at 25°C and at p = 0.1 MPa.

IL	A ± σ	B ± σ	C ± σ	R^2
**[TBP][Sal]** [1]	378.46 ± 147.26	-3.48 ± 0.47	-0.05 ± 0.02	0.7741
**[TBA][Sal]**	213.18 ± 32.15	-1.17 ± 0.15	$(0.23 \pm 0.04) \cdot 10^{-1}$	0.9921
**[TBP][Ac]** [1]	174.71 ± 9.80	-0.49 ± 0.02	$(4.64 \pm 0.58) \cdot 10^{-5}$	0.9975
**[TBA][Ac]**	143.59 ± 2.42	-0.39 ± 0.01	$(4.74 \pm 0.13) \cdot 10^{-5}$	0.9999
**[TBP][Lac]** [1]	133.74 ± 5.41	-0.39 ± 0.01	$(4.39 \pm 0.23) \cdot 10^{-5}$	0.9904
**[TBA][Lac]**	87.26 ± 0.29	-0.27 ± 0.01	$(5.56 \pm 0.06) \cdot 10^{-5}$	0.9999

Table S4. Experimental tie-lines data in percentage weight fraction for the ATPS composed of $(NH_4)_2SO_4$ (X) + IL (Y) + H_2O (Z) at 25°C and 0.1 MPa, and volume ratios (Vr).

IL	ABS composition		IL-rich phases			Salt-rich phases			TLL	Vr
	100·X	100·Y	100·X	100·Y	100·Z	100·X	100·Y	100·Z		
**[TBP][Sal]**	3.28	20.00	0.58	59.84	39.58	4.44	2.80	92.76	57.17	0.25
**[TBA][Sal]**	2.83	20.10	1.61	44.04	54.35	3.23	12.14	84.63	31.93	0.33
**[TBP][Ac]**	23.08	20.03	8.08	41.71	50.21	36.23	0.90	62.87	49.58	0.89
**[TBA][Ac]**	22.99	20.16	6.94	50.94	42.12	31.61	3.64	64.75	53.34	0.80
**[TBP][Lac]**	22.96	20.13	7.08	45.44	47.48	33.70	2.47	63.83	50.54	0.67
**[TBA][Lac]**	22.99	20.17	3.83	50.99	45.18	34.39	1.83	63.78	57.88	0.90

Table S5. Experimental tie-lines data in percentage weight fraction for the ATPS composed of $(NH_4)_2SO_4$ (X) + [TBP][Sal] (Y) + H_2O (Z) at 25°C and 0.1 MPa, and volume ratios (Vr), used during the study the effect of operating parameters.

IL	ABS composition		IL-rich phases			Salt-rich phases			TLL	Vr
	100·X	100·Y	100·X	100·Y	100·Z	100·X	100·Y	100·Z		
**TL1**	3.28	20.28	0.58	59.84	39.58	4.44	2.80	92.76	57.17	0.25
**TL2**	10.07	19.75	0.75	64.70	34.55	14.25	0.15	85.60	65.94	0.38
**TL3**	14.88	20.19	0.84	82.94	16.22	19.44	0.01	80.55	66.96	0.36
**TL3-1**	16.92	10.36	0.84	82.94	16.22	19.44	0.01	80.55	66.96	0.19
**TL3-2**	17.75	6.30	0.84	82.94	16.22	19.44	0.01	80.55	66.96	0.13

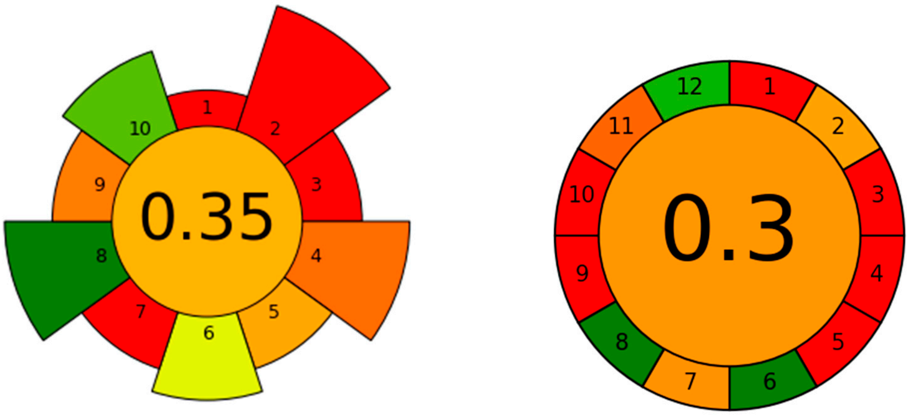

Figure S4. The results of AGREEprep (left) and AGREE (right) assessment of the procedure for pesticide preconcentration from strawberries, with calculations based on data reported in the literature [2].

[1] J. Mušović, D. Tekić, S. Marić, A. Jocić, D. Stanković, A. Dimitrijević, Sustainable recovery of cobalt and lithium from lithium-ion battery cathode material by combining sulfate leachates and aqueous biphasic systems based on tetrabutylphosphonium-ionic liquids, Sep. Purif. Technol., 348 (2024) 127707.

[2] H. Wang, H. Ping, Q. Liu, P. Han, X. Guo, Determination of Pesticide Residues in Strawberries by Ultra-performance Liquid Chromatography-Tandem Mass Spectrometry, Food Anal. Methods, 15 (1) (2022) 85.
